# SUCCESSES IN THE TRANSLATION OF CAREGIVER INTERVENTIONS IN COMMUNITY-BASED ORGANIZATIONS AND HEALTH CARE SYSTEMS

**DOI:** 10.1093/geroni/igz038.1649

**Published:** 2019-11-08

**Authors:** Jinmyoung Cho, Alan B Stevens

**Affiliations:** Baylor Scott & White Health, Temple, Texas, United States

## Abstract

Family caregiving of an older adult has become an essential element of the U.S. health care system, with 83 percent of long-term care provided to older adults coming from family members or other unpaid helpers. With the amount and type of care provided by families expected to increase, caregiving demands should be coupled with community and health care systems-based supports. While scientific research has demonstrated the value of providing education, skills training and support to family caregivers, health care and social service providers do not systematically include these interventions in their services. Thus, for the vast majority of family caregivers, caregiving support services remain extremely fragmented, if not elusive. This symposium provides four examples of how health care systems that frequently see patients with dementia and community-based organizations who provide ongoing supportive services to family caregivers, have adapted evidence-based caregiver interventions into branded service programs. Dr. Jinmyoung Cho will present racial/ethnic comparisons on the impact of community-based implementation of a caregiver education program, REACH-TX. Dr. Leah Hanson will introduce the implementation of Mindfulness-Based Dementia-Care (MBDC) within a health care system. Dr. Christine Jensen will address how caregivers can benefit from evidence-driven programs in health care settings. Lastly, Dr. David Coon will present two different approaches to translation of evidence-based programs through community-based organizations, with CarePRO embedded after completion of a clinical trial and EPIC embedded from the program’s initial pilot phase. The discussant, Dr. Alan Stevens, will highlight the needs of caregivers and support services recognized by all key stakeholders.

